# Randomized Control Study of the Implementation and Effects of a New Mental Health Promotion Program to Improve Coping Skills in 9 to 11 Year Old Children: *Passport: Skills for Life*

**DOI:** 10.3389/fpsyg.2020.573342

**Published:** 2020-10-29

**Authors:** Brian L. Mishara, Sarah Dufour

**Affiliations:** ^1^Psychology Department, Université du Québec à Montréal, Montreal, QC, Canada; ^2^School of Psychoeducation, Université de Montréal, Montreal, QC, Canada

**Keywords:** mental health, promotion, school, evaluation, coping, primary school, children

## Abstract

*Passport: Skills for Life* is a universal, primary school mental health promotion program to increase children’s coping skills. A stratified randomized control study with pretest, post-test and 1-year follow-up included 1,492 3^*rd*^ to 6^*th*^ grade children, from higher and lower socio-economic levels, randomly assigned by school to receive the program or a control group. Implementation and effects were evaluated by questionnaires and focus groups with children, parents and teachers as well as classroom observations. Program activities were well implemented and greatly appreciated, with perceived improvements in resolving conflicts, communicating feelings and coping. Compared to the controls, participants had increased emotional awareness, sustained 1 year later; conceived of more ways to cope in fictitious situations and reported using more, and more useful strategies, sustained 1 year later. Positive Academic Behaviors increased, but were not sustained the following year. This is a promising program to improve coping and emotional awareness that merits further research on its effects.

## Introduction

This article presents the results of the first evaluation of the implementation and the short-term effects of a new mental health promotion program for elementary (primary) schoolchildren, *Passport: Skills for Life* (*PSL*). *PSL* encourages emotional well-being by helping children 9 to 11 years old expand their repertoire of helpful coping skills in order to better equip children to deal with difficulties and stressful problems they encounter in their daily lives, including relationships with peers and parents. This program was conceived as a complement to the widely used and frequently evaluated program for younger 6 to 7 year old children *Zippy’s Friends*, or as a stand-alone program for older age groups. This article begins by presenting the justification and background for developing this new mental health promotion program. We describe the process of creating and validating the program content, and the nature of the program. We then present the methodology and results of a randomized controlled study to evaluate the program’s implementation and short-term effects with children who had no prior participation in school mental health promotion programs. It is hoped that this initial evaluation will lead to further assessments of the impact of *PSL*, including studies of using *PSL* with children who had *Zippy’s Friends* when they were younger.

According to World Health Organization (2018), mental health is a state of well-being in which an individual realizes his or her own abilities, can cope with the normal stresses of life, can work productively and is able to make a contribution to his or her community. *PSL* is based upon a mental health promotion approach which is “conceptualized as an empowering, participative, and collaborative process, which enables peoples to increase control over their mental health and its determinants” (p. 359) (Barry, 2013). The emphasis is on the individual strengths that are present before difficulties occur, before non-adaptive behavioral patterns develop, become embedded and have an important impact on individual functioning (Desjardins et al., 2008).

The WHO has adopted an ecological perspective in understanding the determinants of mental health in adults (Desjardins et al., 2008; World Health Organization, 2018) and in children (Hamel et al., 2001; Patel et al., 2007), where determinants are seen as part of a system, ranging from individual characteristics to social and political environments. A key determinant of mental health in children is the acquisition of capacities to improve their own well-being. Capacities, such as emotion regulation, self-control and coping skills contribute to the ability to successfully confront daily challenges. Many researchers have emphasized the importance of working on key general determinants of mental health, rather than targeting problem-specific determinants, such as violence or bullying, in order to prevent a wider range of problems in children (Greenberg et al., 2000; Browne et al., 2004; Desjardins et al., 2008).

The importance of promoting mental health early in children’s lives has now been scientifically demonstrated and is therefore at the heart of public health policies at the local (Desjardins et al., 2008), national (Public Health Agency of Canada, 2014), and international levels (World Health Organization, 2018). Mental health promotion in children is based upon two main arguments. The first argument is pragmatic: since children are already exposed to numerous stressful situations in their daily lives, they need to have a range of individual capacities to deal with and adapt to these situations. For example, children may be confronted with alcoholism or depression in their family, or the divorce of their parents; some may have a chronic illness that seriously affects their lives. At school, children may have academic difficulties or experience rejection by their peers (Wolchik and Sandler, 1997). The extent of their individual capacities will determine how well they will cope with the challenges and difficulties they face and the impact of those experiences on their lives. The second argument is developmental: childhood is a critical period in the development of mental health (Desjardins et al., 2008; Weare and Nind, 2014), thus early interventions are likely to generate the greatest benefits (Huppert, 2014). Studies have shown that the early development of coping skills helps children deal with the difficulties and problems they will encounter throughout life (Boekaerts, 1996). Therefore, the development of individual coping skills is a legitimate and recognized target for actions in mental health promotion with children. Ideally, learning coping skills should be combined with interventions at other systemic levels, such as improving educational practices and increasing the quality of children’s social environment.

School is an ideal setting for mental health promotion in children: it has a significant impact on children’s development, since they attend school on a daily basis (Barry, 2013; O’Mara and Lind, 2013; Weare and Nind, 2014). This vision expands upon the traditional educational role of schools to include the potential of schools to influence children’s health and well-being (Weare, 2010). There are now an increasing number of school-based initiatives in mental health promotion, many of which have been scientifically evaluated. These initiatives can fall into either of two categories: First, there are learning programs that teach individual skills and competencies; they refer explicitly to mental health promotion or to related concepts, such as social and emotion education, emotional intelligence or life skills. The second, more recent approach is whole school programs, which aim “to enable schools to develop learning and working environments that promote better health for everyone” (p. 206) (O’Mara and Lind, 2013). In their analysis, Weare and Nind (2014) concluded that school-based mental health promotion interventions have clear positive impacts on well-being, positive mental health, and social and emotional learning, with very few examples of adverse effects. When implemented effectively, well-designed school programs promoting mental health and well-being can result in multiple benefits for children (Weare, 2010; Barry, 2013; Weare and Nind, 2014). However, even though these interventions are clearly promising, their evaluations often have limitations, including lack of assessment of the perceptions of the children involved in the programs, inconsistent results, and methodological shortcomings, which limit the conclusions we may draw from the evaluations (Weare, 2010; O’Mara and Lind, 2013).

*PSL* was developed as part of an initiative in mental health promotion for children financed by the Public Health Agency of Canada. This project was inspired by the international mental health promotion program for younger children, *Zippy’s Friends (ZF)*. *PSF* was developed to be used in schools either independently, or as a follow-up to the ZF program. *ZF* has the primary goal of developing coping skills and social abilities in young children aged 6–7. *ZF* is currently used in 32 countries around the world, with over 1.6 million children having participated to date. Several evaluations conducted in different countries have shown that *ZF* improves coping and social skills (e.g., Mishara and Ystgaard, 2000; Monkeviciené et al., 2006; Mun, 2008; Clarke and Barry, 2010; Dufour et al., 2011; Holen et al., 2012). Schools that use *ZF* have often asked for a similar program for older more mature children, in order to sustain their acquired skills and to further broaden their range of coping and social abilities at a more sophisticated level. PSL shares the main objective of ZF of increasing children’s repertory of helpful coping skills. Secondary objectives include improving children’s social skills of recognizing and communicating feelings; as well as accepting, seeking and offering help. Although *PSL* was conceived as a complete stand-alone program, combining *Zippy’s Friends* and *Passport: Skills for Life* is envisioned to provide an even better opportunity for expanding and consolidating the acquisition of helpful coping skills.

The final version of the *PSL* program that was evaluated in the present study is the result of a rigorous process of perfecting the activities over 5 years. Before beginning to develop the program, focus groups were conducted with 88 key informants, including educators, parents, public health experts and representatives of community organizations. These 15 focus groups allowed for a better understanding of perceptions of the relevance of developing this program, the objectives to pursue, the preferred pedagogical methods, as well as potential obstacles and constraints to take into consideration. Subsequently, an intensive 3-day advisory workshop was organized, with 14 participants from schools, community organizations and the Quebec Public Health Institute (INSPQ). This workshop validated the objectives, the format (length, type of activities, pedagogical approach and methods, etc.) and was used to brainstorm on the nature of the activities to be developed.

A pilot program was then created, tested and evaluated with 15 classes in seven schools, with 269 children. The following year, based upon the experiences with the pilot program, a Beta version of PSL was developed and its implementation and perceptions of the program were evaluated in summer camps with 1,008 children. The activities where then adjusted based upon the pilot testing and the complete Beta version of *PSL* was created. The implementation of the Beta version was evaluated in detail and instruments for assessing its effects were tested with 488 children from 22 classes in six schools, and compared with a Control Group of 347 children from 16 classes in five schools. Following the promising results of the evaluation of the Beta version (unpublished), and the assessments of the implementation, the appreciation and participation in each component and activity and the assessments of effects, modifications to the Beta version were made. All of the activities that were modified were then tested in summer camps with 662 children. Based upon the results of those tests, the “final” version of *PSL* was made.

During the various stages in the development of PSL, activities were modified or replaced when teachers reported difficulties in conducting the activity in class, teachers reported a lack of positive impacts of the activity, the activity had a low rate of participation by children, children did not accomplish the tasks involved in the activity as planned, or there was an absence of high levels of appreciation of the activity by children, teachers or parents. Also, sometimes additional activities on a topic were added to reinforce a program goal. For example, when it was observed that the secondary program goal of increasing asking for help and offering help had not been achieved in the evaluation of the Beta version of the program, activities were added on helping others and using help. In addition, a “Helping Thermometer” was added which awarded points to advance in the program’s much appreciated Dragon’s Path game whenever seeking or giving help was observed by the teacher during *PSL* sessions in order to increase awareness of seeking, accepting and offering help.

This paper reports on the evaluation of the implementation and the effects of this final version, which incorporates all improvements to the activities and material based upon the preceding stages of the program development. The goal of this evaluation is to determine if *PSL* can be easily implemented as planned in a successful manner, and if this program has the desired effects on participants. The main goal of the program is to improve children’s coping skills, and this is the main outcome variable of interest. In addition, we explored if the program may improve children’s emotional awareness, as well as some academic and social skills.

## Materials and Methods

### Program Description

**FIGURE 1:**
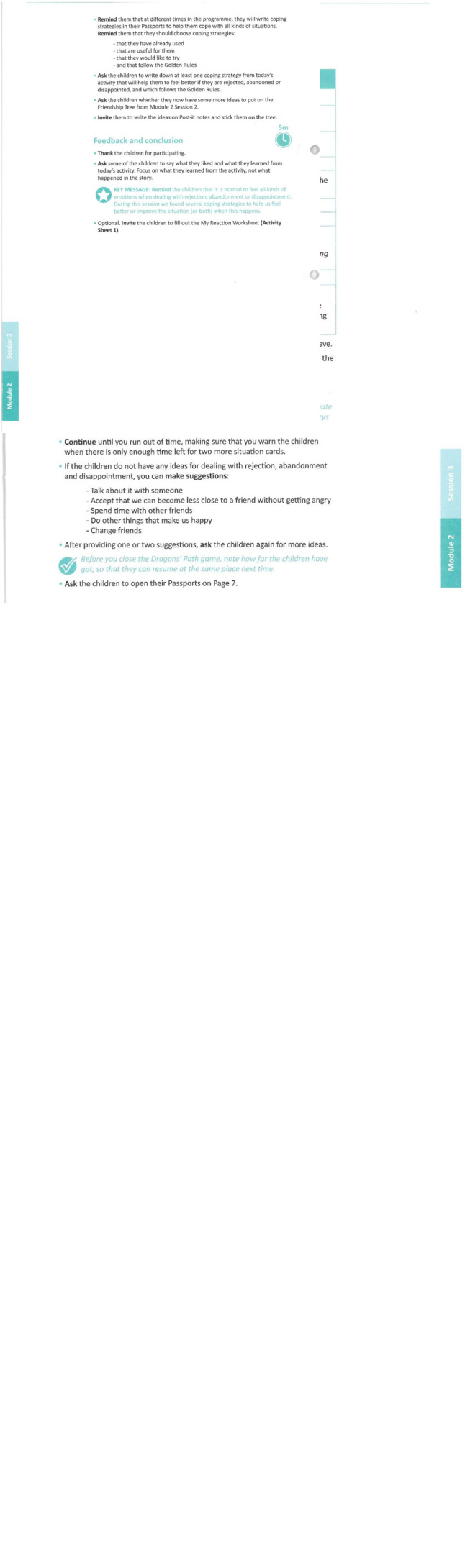
Example of the teacher’s manual instructions for a session of the *Passport Skills for Life* program.

*PSL* includes a short introductory session, followed by 17 sessions (55 min each) conducted in class by their teachers, who receive a day of training before the program begins and an additional 4 h of training halfway through the program, and for whom a resource person is available to consult if needed. At the beginning of each session, children are asked to read an entertaining comic strip created to illustrate the theme of the session. Then, children engage in activities to identify, experiment with and evaluate the usefulness of different coping skills in real life situations children may experience. An example of a Session may be seen in Figure 1, which shows the instructions to teachers for one of the Sessions.

In each Session, the story the children read in the weekly comic book introduces the topic, and the activities in the session involve understanding the topic as well as identifying helpful coping skills the children may use in their personal lives. Children note coping skills they learned in their personal “Passport” at the end of each session. For example, in Module 4 Session 1 the topic of coping with “Unfair Situations Around Me” is introduced by the comics story in which the main characters are frustrated and upset when the parents of a young “giant” forbid him from going to a music concert with friends because he is “too young.” The friends offer to help the young giant find ways to deal with the situation. Then, the children have an activity where they discuss if the parents were unfair, and the difference between being unequal and unfair. Their next activity in the session involves role playing situations where children feel things are unfair and suggesting various ways to cope with the situation that can make the children feel better or improve the situation, without harming anyone. For each coping skill identified, they move ahead one block in the classroom game, the Dragon’s Path (which is described in Figure 1).

The program has five modules, each with three sessions (see Table 1), followed by a summary session and a celebration where children receive diplomas. Seven activities are provided to parents to conduct at home with their children, for each of the five modules and the final sessions. The turnkey material provided to teachers with step by step instructions includes comics to hand out each week, and all the other material needed for each activity, copies of parent information bulletins and home activities sent to parents for each module, summary posters and illustrations, individual “Passport” booklets with reminders and places for children to note their coping strategies, an illustrated “Coping Kit” box each child brings to the final session, and a giant game board used by the class throughout the program. The detailed Teacher’s Guide includes tips on integrating the sessions’ concepts with academic school activities. A web site has additional activities for children, parents, teachers, and school personnel^1^. Throughout *PSL*, no specific coping skills are said to be good or bad. Rather, children are encouraged to help each other identify many potential strategies to cope with each situation. Then, children individually assess the usefulness of the strategy for improving the situation or making them feel better, while not harming anyone. Competition is discouraged and giving and asking for help are practiced. More details of *PSL* may be found at https://www.partnershipforchildren.org.uk/what-we-do/programmes-for-schools/passport.html and www.passportskillsforlife.ca.

**TABLE 1:** Content of the Modules and Sessions in *Passport: Skills for Life.*

Module 1 – Emotions	Session 1 – Valuing our differences and similarities
	Session 2 – Understanding and expressing our emotions
	Session 3 – Recognizing other people’s feelings
Module 2 – Relationships and helping each other	Session 1 – Helping each other and coping skills
	Session 2 – Friendships
	Session 3 – Challenges in friendships
Module 3 – Difficult situations	Session 1 – Dealing with frustration
	Session 2 – Dealing with stress
	Session 3 – Dealing with conflict
Module 4 – Fairness, justice and what is right	Session 1 – Unfair situations around me
	Session 2 – Dealing with bullying
	Session 3 – Dealing with unfair and unjust situations
Module 5 – Changes and loss	Session 1 – Big news: coping skills for dealing with change
	Session 2 – Dealing with loss
	Session 3 – Helping each other in difficult situations
Final review session	The coping kit – Review
Party session	Celebration and distribution of diplomas

### Participants

Schools were recruited to participate in this study and were told that all the participating schools would be randomly assigned to either an Experimental Group, who would receive the program and participate in the evaluation, or the Control Group, who would participate in the evaluation, would not receive the program during the current academic year, but would receive the program at no cost after completion of the study. The schools were classified according to their community’s socio-economic level using the “impoverishment” index published by the government of Quebec for each school district, classifying schools as being in relatively more impoverished and relatively non-impoverished communities, and within each group, schools were randomly assigned to the Experimental or Control Groups. Although all the schools had a heterogeneous population of children from various socio-economic backgrounds, this crude categorization was used to help ensure that both the Experimental and Control Groups did not differ significantly in terms of their socio-economic level.

The Experimental Group consisted of 44 classes in 12 schools with a total of 872 students, and the Control Group had 46 classes in eight schools with a total of 991 students. In the Experimental Group, 767 of the parents (86%) returned signed Consent Forms authorizing their children to participate in the research and 971 of the parents (98%) in the Control Group returned signed consent forms. No parents refused participation on the returned consent forms, but children whose parents did not return the forms were not included in the evaluation. Of the 1,863 children in both groups whose parents consented to their participation, 1,492 constituted the final sample for the evaluation of changes between the pre-test and post-test (666 in the Experimental Group and 826 in the Control Group), since 246 children were either absent for the pre-test or the post-test assessment, or changed schools or classes during the course of the study.

There were 315 boys (47%) and 349 girls (52%) in the Experimental Group; and 406 boys (49%) and 417 girls (50%) in the Control Group. The distribution and percentage by school year (3^*rd*^, 4^*th*^, 5^*th*^, and 6^*th*^ grade) and socio-economic level of the schools may be seen in Table 2.

**TABLE 2:** Characteristics of participants in the evaluation of *Passport: Skills for Life* [Number of children (%)].

		Experimental group	Control group	Total
Sex	Boys	315 (47.3%)	406 (49.2%)	721 (48.3%)
	Girls	349 (52.4%)	417 (50.5%)	766 (51.3%)
	Missing data	2 (0.3%)	3 (0.3%)	5 (0.4%)
	Total	666	826	1492
Socio-economic level	Schools from non- impoverished areas	215 (35.4%)	308 (41.0%)	523 (38.8%)
	Schools from impoverished areas	393 (64.6%)	443 (59.0%)	826 (61.2%)
	Missing data	58	75	133
Grade level	3^rd^ year	159 (23.9%)	232 (28.2%)	391 (26.4%)
	4^th^ year	173 (26.0%)	271 (33.1%)	444 (29.9%)
	5^th^ year	181 (27.2%)	184 (22.4%)	365 (24.5%)
	6^th^ year	136 (20.6%)	134 (16.3%)	270 (18.2%)
	Mixed-level	15 (2.3%)	-	15 (1.0%)
	Missing data	1	4	5

Of the 1,492 who were present for the pre-test and post-test assessments, 1,095 were also evaluated in the same school 1 year after the end of the program, the others having changed schools or not being in class at the time of the 1 year follow-up. The missing students included 136 who were in the 6^th^ grade during the study and had changed to a secondary school the following year. Ethical approval was obtained from the Ethics Review Committees of both universities where the researchers are affiliated, as well as the school commissions that also had ethics review boards. Participants did not receive any compensation for their participation.

### Measures

#### Evaluation of the Implementation

Teachers completed an evaluation of the teacher training sessions. After conducting each of the 17 program sessions, teachers were asked to complete a questionnaire indicating their ease in conducting that session, their appreciation of the session, its perceived impact on children, and suggestions for improvements. After each session, children were asked to indicate their appreciation of the session by coloring or circling pictures to indicate one of four levels of how much they liked the session, with space to include any comments. Parents were given an evaluation form to fill out and return after each of the seven parent–child home activities, indicating how useful they felt the activities were on a four-point scale, what they liked and disliked about the activity, with a space to include any comments.

Observations of 89 complete sessions were conducted independently by two trained observers (graduate students in psychology) in each class. A third observer, the first author of this paper, was also present to independently complete observations in 11 sessions. Observations were conducted in all 44 classes, with observations spread across all 16 sessions. In addition to these 89 observations, observers also attended 8 classes during the 17^th^ session, which is a party where children received program diplomas. However, because there is no didactic content, systematic ratings of the 17^th^ session were not conducted. Each teacher was observed at least once and each session was observed at least twice. Observers rated the frequency of occurrence of different behaviors on a four-point scale (never, sometimes, often, always). They counted the total number and percentage of boys and girls participating in each of the two class activities in each session, the extent to which the teacher ran the session in accordance with the implementation manual and the principles of the program, and made other observations about the enthusiasm of participants, the general climate during the program and they noted any behavioral incidents observed. After completing their independent observations, the observers compared their ratings, any discrepancies were discussed, and agreement was reached. Overall, before discussion to reach agreement, there were very few discrepancies in ratings, with minor disagreement in less than 3% of ratings in all observations with the exception of the category, “The teacher judged the students’ responses.” During the first 3–4 observations, some raters considered a remark praising a response to be a “judgment,” (e.g., saying “Very good, Johnny”), even when the teachers consistently praised all children whenever they responded, regardless of the nature of the response. Judgment was then defined in the observations as referring to communication by the teacher to a student that a specific suggestion by the student about how to cope was good or bad, effective or ineffective, rather than including praising a student for having responded without reacting to the content of the response. In *PSL* teachers are supposed to not judge any ways of coping that children suggest as part of the *PSL* activities. Teachers are instructed to always ask the children to determine by themselves whether or not a coping strategy should be retained, by assessing if they believe the strategy would help improve the situation, make the you feel better, or both, and not harm anyone. After the redefining of this category, there were only three minor disagreements in subsequent observations.

After the program was concluded, focus groups were conducted in the schools, lasting between 1 h and 90 min: 14 focus groups with teachers (*n* = 53), two focus groups with parents (*n* = 8) and seven focus groups with children (*n* = 61). These groups began with open-ended discussions on the impact of the program, and then included prompt questions where they were asked what they thought about the activities. They were then asked if they had any suggestions for improvements. The teacher groups were asked the same questions. Teachers were also asked if the training and support they received in conducting the program was adequate, they were asked what was their perceptions of the impact of the program on children, the class and school environments. Focus group sessions were transcribed and analyzed by two research assistants skilled in qualitative analyses, using NVivo, to identify thematic content.

#### Evaluation of the Effects

The following quantitative measures were used to compare Experimental and Control Groups in Pre-Test, Post-Test and One Year Follow-Up.

#### Emotional Awareness

Recognition of feelings or ‘emotional awareness’ was assessed based upon responses by children to four written descriptions of problem situations that children their age often experience: being a victim of bullying, witnessing bullying, experiencing unfair parental restrictions and the family moving away to another place. A description of each of these situations was read aloud, and then the children were given 3 min to write answers to two things about each situation: (1) how the child experiencing the problem felt, and (2) what the child in the situation could do (which was used to assess coping strategies, see below). In Canada, children in primary school grades four to six almost universally have the necessary writing skills to name basic feelings in response to questions such as these. The answers to the feeling questions were scored according to the Levels of Emotional Awareness Scale (LEAS-C) (Barchard et al., 2011) on a scale from 0 to 5, with 0 indicating no awareness or mentioning of any emotions, and 5 indicating description of more than one discrete emotion. Two research assistants were trained on coding the written responses and discussed any discrepancies until near perfect agreement was obtained.

#### Coping

Because of the primary importance of coping as a key element in the program and difficulties is assessing coping in children (Spirito, 1996), coping was measured on an exploratory basis using four separate instruments which each assess aspects of how children cope in a somewhat different manner:

##### Coping in hypothetical situations

In each of the four hypothetical problematic situations used to assess emotional awareness, children were also asked to describe what the character could do in that situation. The coping strategies described in the open-ended questions were classified by two independent research assistants in one of 13 categories which included all coping strategies that were present in responses by the children. Since one of the coping strategies, ‘physical violence toward others’ is considered undesirable, this strategy was not included in the calculations of the total number of helpful coping strategies used. The research assistants were trained on coding the written responses and discussed any discrepancies until near perfect agreement was obtained.

##### Draw and write

With this instrument, which was previously used to assess the effects of *ZF* with much younger children with significant results (Clarke and Barry, 2010), the children were asked to think of a situation when they felt sad and make a drawing of the situation in the first frame on a paper. The children were then asked to write a one-sentence description of the situation below the drawing. After a few minutes, the children were instructed that after they finish their drawing of the situation, they should draw, in the second frame, what they did to improve the situation or to feel better. They were also asked to write a sentence below the second drawing describing what they did to improve the situation or to feel better. The coping strategies that were written by the children under the drawings were classified using the same categories as in the Coping in Hypothetical Situations.

##### Kidcope

In the Kidcope (Spirito et al., 1988), we asked children to ‘think of the problem that bothered you the most in your relationship with other children recently’ and then asked them to answer whether or not ‘you used these ways to deal with the situation,’ for each of 16 coping strategies. Children were also asked, for each coping strategy, to rate ‘How much did it help?’ on a three-point scale: Not at all, A little or A lot. Spirito et al. (1988) reported test–retest reliabilities over 3 to 7 day periods from 0.41 to 0.83.

##### Children’s Coping Questionnaire (CCQ)

In the Children’s Coping Questionnaire (CCQ), which was developed and validated in a doctoral dissertation (Fedorowicz, 1998), we asked children, just as with Kidcope, ‘to think about the problem that bothered you the most in your relationships with other children recently.’ However, rather than asking what coping strategies they used, the CCQ asks children a slightly different question, to choose on a four-point scale (never, a little, pretty much, a lot) ‘the answer that best describes what you do when the problem arises’ for 24 coping strategies.

#### Social and Academic Skills: Social Skills Rating System

The Social Skills Rating System (SSRS) (Gresham and Elliott, 2008) is a questionnaire used to describe children’s social behaviors with both a 34-item self- administered children’s version and a 30-item version in which the teacher fills out the questionnaire for each child. For each item the child and teacher are asked to indicate on a three-point scale (never, sometimes, very often) how frequently they engaged in each behavior. The SSRI showed strong psychometric properties in terms of internal consistency and test–retest reliability (Gresham and Elliott, 2008; Gresham et al., 2011). Median scale reliabilities are in the mid and upper 0.90s and median subscale reliabilities are in the high 0.80s for the Teacher Form and near 0.80 for the Student Form.

## Results

### Implementation of the Program

The objectives of the evaluation of the implementation of the program were to determine if the program was conducted as planned, and if the teachers, children and parents who participated in the program appreciated each of the program’s activities. We also explored why they appreciated the program and if there were some activities or program characteristics that were perceived as being in need of modifications.

The teachers’ evaluation of the initial training and the mid-term training sessions indicated high levels of appreciation of all aspects of the training, with 96% ‘completely’ or ‘generally’ agreeing to all statements about the positive aspects of the training. In fact, all teachers but five were always in agreement with all statements about the positive aspects of the training, and those five only disagreed somewhat with the statement that the duration of the training was sufficient.

The teachers completed 756 evaluation forms after conducting sessions, with between 30 and 49 forms completed and returned per session. These forms confirmed that all teachers felt they were able to conduct the program as planned; there were many positive comments about the perceived benefits of the program; and the teachers particularly appreciated the clear manner in which the activities were explained and the ease in which the program’s principles could be applied to other situations and activities in school. Negative comments included a few teachers who found it difficult to complete all the activities within 55 min, some difficulties with the youngest children (in grade 3) requiring additional explanations, and some suggestions to change the wording or content of role play activities for the grade six children. All of the improvements suggested by teachers were made.

Thematic analyses of focus groups indicated that the teachers generally had an extremely high level of appreciation for the program and their experience in conducting it (‘exceeded my expectations’). They liked the turnkey organization of the material, the visual quality of the posters and comics, the ability of the program to elicit discussions of feelings and important issues in children’s lives, and its concrete activities.

The children completed a total of 14,364 appreciation forms at the end of the sessions. On a four-point scale indicating their appreciation for the sessions (1 = not appreciated, 2 = somewhat appreciated, 3 = appreciated, 4 = appreciated at lot), appreciation levels varied over the sessions, ranging from 2.66 and 2.80 on the last two sessions to 3.36 in the first session. The most frequent comments on the last two session appreciation forms was disappointment that the program was to end soon. Girls appreciated seven of the 16 sessions significantly more than boys, but the mean differences were not great (never more than 0.2 on average, on the four-point scale). Children in schools in communities with lower socio-economic levels had significantly higher appreciation levels for 11 of the sessions, but again the mean differences were not great, the greatest mean difference being 0.28 points.

In focus groups, besides expressing great appreciation for the program, children said they particularly enjoyed the comics, the activities where they moved around, and being able to freely express their feelings without judgment.

The parents completed and returned 1,078 evaluation forms after conducting activities with their child. They indicated that the at-home activities lasted between five and 20 min each. 82% indicated that they were in ‘agreement’ or ‘completely in agreement’ with the statement that the activities were helpful to their child, 16% felt the activities were ‘somewhat helpful’ and 2% felt the activities were not helpful. In focus groups, although there is a strong possibility of selection bias with only eight parents accepting the invitation to participate in a focus group, it is interesting to note that they all felt the program allowed them to better know what their children thought about important issues and how to better cope with problems.

Table 3 summarizes major findings from the 89 observations of sessions concerning conformity of the implementation. All of the 89 sessions observed were generally conducted as planned. However, during the first eight weeks of the program, over half of the teachers (53.7%) failed to encourage children to ask for help and to offer help to others. Asking for and offering help is encouraged in *PSL*, and a “helping thermometer” was introduced to encourage these behaviors by giving points to advance on the much appreciated Dragon’s Path game for helping behaviors that the teacher observes. Based on these initial observations, the mid- program training after Session 8 was revised to greatly emphasize the need for teachers to encourage help seeking and offering help. After participating in the revised training, the teachers were observed to encourage children to ask for and offer help in 94.9% of the sessions “often” or “always.” The percentage of children actively participating in each individual activity ranged from 32 to 100%, with a median of 90% participation; 79% of the activities had over 80% active participation.

**TABLE 3:** Classroom observations (*N* = 89 sessions) for each of the activities (2 per session, total ratings = 178), before and after mid-program training sessions.

Measure	Sessions 1–8 before mid-program training *N* (%) Never or rarely	Sessions 1–8 before mid-program training *N* (%) Often or always	Sessions 9–16 before mid-program training *N* (%) Never or rarely	Sessions 9–16 before mid-program training *N* (%) Often or always	All sessions 1–16 N (%) Never or rarely	All sessions 1–16 *N* (%) Often or always
Teacher uses material as planned for session	1/59* 1.7%	58/59* 98.3%	0/118 0.0%	118/118 100.0%	1/177* 0.6%	176/177* 99.4%
Teacher has mastery of the content	4/60 6.7%	56/60 93.3%	0/118 0.0%	118/118 100%	4/178 2.2%	174/178 97.8%
Teacher is enthusiastic conducting activities	4/58** 6.9%	54/58** 93.1%	2/118 1.7%	116/118 98.3%	6/176** 3.4%	170/176 96.6%
Teacher conducts session as indicated	7/60 11.7%	53/60 88.3%	4/118 3.4%	114/118 96.6%	11/178 6.2%	167/178 93.8%
Teacher judges children’s responses	57/60 95.0%	3/60 5.9%	111/116** 95.7%	5/116** 4.3%	168/176** 95.5%	8/176** 4.5%
Teacher encourages children to ask for and offer help	29/54*** 53.7%	25/54*** 46.3%	6/118 5.1%	112/118 94.9%	35/172*** 20.3%	137/172*** 79.7%
Children pay attention to teacher	5/58** 8.6%	53/58** 91.4%	4/118** 3.4%	114/118 96.6%	9/176** 5.1%	167/176** 94.9%
Children are enthusiastic in their participation	6/60 10.0%	54/60 90.0%	5/116** 4.3%	111/116** 95.7%	11/176** 6.3%	165/176** 93.7%

*Data for 1 activity missing. **Data for 2 activities missing. ***Data for 6 activities missing.

Overall, appreciation levels were very high for the program in general and each of the specific components, for children, parents and teachers. The program could be easily implemented as planned by teachers who have participated in the *PSL* training activities. Some teachers had difficulties completing some of the sessions in 55 min. However, those teachers were able to extend the session by 5 to 10 additional minutes when needed, and the difficulties completing activities within 55 min were most common during the first few sessions they taught. Because of our finding that the youngest third grade children, age 9 on average, required additional explanations to understand all the activities, this suggests that the *PSL* is best suited for children starting in grade 4, age 10 and over. There were no indications that the program needs further modifications. The teachers, children and parents particularly liked the quality of the material and the nature of the activities. Teachers attributed the ease of conducting the program to the training they received and the turnkey organization of how to proceed, with all material furnished in advanced and organized to be easily used in the classroom.

#### Qualitative Outcome Measures: Perceived Effects Reported by Teachers, Parents and Children

Effects of the program were evaluated by quantitative assessments using standardized measures (reported in the next section), as well as qualitative indicators of perceived effects reported by teachers, parents and children. The perceived changes are both a potential indication of the behavioral effects of *PSL*, and an essential indicator of the potential for uptake of the programs in schools. If children, parents and teachers feel that there are obvious benefits from the program, they are much likely to support continuing to use *PSL* in their school and they may help convince others to adopt the program elsewhere. We assessed perception of *PSL* and its effects by conducting focus groups.

In the focus groups, participants were asked to describe any changes perceived by them following participation in *PSL*. Qualitative analyses were conducted on transcripts of the focus groups discussions by a research associate who was not present during the focus groups, using NVivo. Themes that were identified included: teachers said that children dealt with conflicts better after the program and they had to intervene less often. Teachers said they learned new coping strategies that they used in their personal lives. They also felt they developed a better and more intimate relationship with their students.

Children said the program helped them learn new ways to handle conflicts and frustrating situations. They said they learned to calm down, make compromises, respect what others said, ask adults for help, write feelings in a journal and communicate their feelings to adults. According to parents, *PSL* was useful for their children, the program and home activities permitted discussions of important issues for the first time, and some said their child denounced a situation involving bullying.

In general, children, teachers and parents reported positive benefits from participation in the program, including improvements in the main goal of *PSL*, using new effective coping strategies to deal with problems experienced in daily life.

### Quantitative Effects Based Upon Comparisons Between Experimental and Control Groups Using Standardized Measures

Four different quantitative measures of coping were assessed comparing children who in the Experimental Group who participated in *PSL* with a control group, in order to verify, using standardized quantitative measures, if the positive changes in coping reported in qualitative data are confirmed. In addition, we assessed emotional awareness, one of the essential skills taught in *PSL*, which is considered to be essential for effective use if helpful coping strategies. Although improvements in social and academic skills are not specific objectives of *PSL*, we included assessments of these variables on an exploratory basis, since teachers reported improvements in classroom behaviors and social skills. Also, research of the *Zippy’s Friends* program for younger first grade children had found improvements in social skills (Mishara and Ystgaard, 2000).

#### Coping

##### Coping strategies envisioned in fictitious situations

Not all children indicated a coping strategy in response to the four fictitious situations. The 191 children who did not indicate any coping strategies were excluded from these analyses, since we cannot know if they could not think of a strategy or simply did not respond to this question. There was a significant interaction between Group (Experimental vs. Control) and Time (pre–post) in repeated measures MANOVA tests examining changes in the number of strategies reported in the four situations [*F* = 3.09 (1,1300), *p* < 0.05]. There was an overall increase in the number of strategies in the Experimental Group compared to a small decrease in the Control Group, and there were no significant interactions with sex or the socio-economic level of the school.

When the 1 year follow-up date were included, the number of children with complete data at all three times of measurement, excluding those who did not indicate any coping strategy in at least one of the times, resulted in a total sample size of 617 children. MANOVA tests indicated a significant interaction between Group (Experimental/Control) and Time (pre-test/post-test, follow-up) [*F* = 4.09 (2,616), *p* < 0.05], with increases in the Experimental Group and no significant changes in the Control Group.

##### Draw and write

Comparisons on the number of coping strategies reported by the children in their written descriptions of the drawings indicated a significant interaction between Group (Experimental/Control) and Time (pre- test/post-test) [*F* = 4.78 (1,1301), *p* < 0.03], with the Experimental Group reporting more strategies in post-test and the Control Group reporting no changes in the number of strategies. There were no significant interactions with sex or the socio-economic level of the school.

When the 1 year follow-up was included in analyses, repeated measures MANOVA analyses of the number of coping strategies reported indicated a significant interaction between Time (pre-test, post-test, 1-year follow-up) and the Group (Experimental/Control) [*F* = 4.61 (2,1083), *p* < 0.01], with *post hoc* (Tukey, Scheffe and Bonferroni) tests indicating significant increases in the Experimental Group from the pre- test to the combined post-test and 1 year follow-up (*p* < 0.01) and no significant difference between the post-test and the 1 year follow-up in the Experimental Groups. There was a significant increase in the number of strategies used in the Control Group from pre-test to combined post-test as well (*p* < 0.03), but there was a significantly greater increase in the Experimental Group post-tests, when compared to the increase in the Control Group (*p* < 0.03).

##### Kidcope

There was a significant interaction between the Group (Experimental/Control) and Time (pre-test vs. post-test) [*F* = 3.74 (1,1490), *p* < 0.05], and no significant interactions with the socio-economic level of the school or the sex of the children, in the total number of different coping strategies used. In the Experimental Group the children increased from a mean of 6.28 (*SD* = 3.67) strategies before participation in the program to a mean of 7.16 (*SD* = 3.34) strategies after participation. The number of strategies used by the Control Group decreased over the same period (pre-test mean 7.17, *SD* = 2.89; post- test mean 6.67, *SD* = 2.82). When the overall usefulness of strategies was calculated by summing up the reported helpfulness of strategies used by each child, there was a significant interaction between Group and Time [*F* = 6.64 (1,1490), *p* < 0.009], with the mean usefulness scores for the Experimental Group increasing from 11.18 in the pre-test (*SD* = 7.29) to 13.02 in the post-test (*SD* = 6.98) and a small decrease in the Control Group (pre-test 14.67, *SD* = 7.03; post-test 13.64, *SD* = 6.77). Unfortunately, Kidcope was not included in the 1-year follow-up.

##### Children’s Coping Questionnaire (CCQ)

When only the pre-test and post-test scores were considered, there were no significant changes on the CCQ in the overall number of strategies used in the Experimental Group from the pre-test (mean 60.41, *SD* = 10.30) to the post-test (mean 60.17, *SD* = 9.62), or in the Control Group (pre-test mean 58.56, *SD* = 10.52; post-test mean 57.88, *SD* = 8.73).

However, when the 1-year follow-up was included in the analyses, there was a significant interaction between Group (Experimental/Control) and Time (pre- test/post-test and 1-year follow-up) [*F* = 5.78 (2,1093), *p* < 0.02], and no significant interactions with the socio-economic level of the school [*F* = 0.81 (2,1073), NS] or the sex of the children [*F* = 0.15 (2,1089), NS]. The Experimental Group had a small mean overall increase in the total number of different coping strategies used from the pre-test to the combined post-tests (60.31 to 60.38), while the Control Group had a decrease (60.38 to 58.66). There were no significant interactions with sex (*F* = 0.15, NS) or with level of socio-economic level of the school (*F* = 0.81, NS).

#### Emotional Awareness

When all four situations are considered together in MANOVA analysis, comparing Experimental and Control Groups on the Emotional Awareness scores, there is a significant interaction between Pre–Post and Group [*F* = 29.33 (1,1491), *p* < 0.0001] with a mean increase from 3.01 (*SD* = 0.54) to 3.23 (*SD* = 0.45) in the Experimental Group compared to no significant change (mean 3.11, *SD* = 0.56 to mean 3.05, *SD* = 0.44) in the Control Group, with no significant interaction with sex [*F* = 1.52 (1,1489), NS] or socio-economic level of the school [*F* = 0.53 (1,1356), NS] (see Table 4).

**TABLE 4:** Changes in quantitative measures in Experimental and Control Groups from Pre-Test to Post-Test and One Year Follow Up.

Measure	Group	*N*	Pre-test mean (SD)	Post-test mean (SD)	One year follow-up mean (SD)	Significant Results Group X PrePost	Significant Results Group X PrePost 1 year FollowUp,	Significant Interaction with sex, school socio- economic and grade
Emotional	Experimental	666	3.01 (0.54)	3.23 (0.45)		*F* = 29.33		None
awareness	Control	826	3.11 (0.56)	3.05 (0.44)		(1,1491),		
						*p* < 0.0001		
	Experimental	465	3.12 (0.37)	3.30 (0.36)	3.22 (0.37)		*F* = 17.53	None
	Control	630	3.11 (0.38)	3.18 (0.37)	3.09 (.44)		(2,1093),	
							*p* < 0.001	
Coping in	Experimental	555	1.01 (0.43)	1.08 (0.41)		*F* = 3.09		None
fictitious	Control	746	0.98 (0.44)	0.89 (0.42)		(1,1300),		
situations						*p* < 0.05		
	Experimental	271	1.09 (0.36)	1.18 (0.37)	1.17 (0.36)		*F* = 4.09	None
	Control	347	1.01 (0.33)	0.97 (0.31)	1.03 (0.29)		(2,616),	
							*p* < 0.05	
Draw and	Experimental	556	1.01 (0.44)	1.17 (0.49)		*F* = 4.78		None
write	Control	746	1.02 (0.46)	1.03 (0.44)		(1,1301),		
coping						*p* < 0.03		
	Experimental	462	1.03 (0.36)	1.41 (0.33)	1.21 (0.33)		*F* = 4.61	None
	Control	623	1.00 (0.30)	1.02 (0.34)	1.04 (0.34)		(2,1083),	
							*p* < 0.01	
Kidcope number of strategies	Experimental	665	6.28 (3.67)	7.16 (3.34)	Not used	*F* = 3.74	Not used	None
	Control	826	7.17 (2.89)	6.67 (2.82)		(1,1490), *p* < 0.05		
Kidcope usefulness of strategies	Experimental Control	665 826	11.18 (7.29) 14.67 (7.03)	13.02 (6.98) 13.67 (6.77)	Not used	*F* = 6.64 (1,1490), *p* < 0.009	Not used	None
Children’s Coping Questionnaire	Experimental Control	665 826	60.41 (10.30) 58.56 (10.52)	60.17 (9.62) 57.88 (8.73)		N.S.		None
	Experimental Control	465 630	59.31 (10.28) 60.38 (10.54)	60.42 (12.18) 58.62 (8.94)	62.34 (10.61) 57.04 (9.23)		*F* = 5.78 (2,1093), *p* < 0.02	None
SSRS Teachers Positive Academic Behaviors	Experimental Control	651 825	12,42 (3.87) 12.28 (4.31)	12.73 (3.97) 12.20 (4.38)		*F* = 5.35 (1,1475), *p* < 0.02		None
	Experimental Control	465 628	12.45 (3.85) 12.37 (4.08)	12.66 (3.91) 12.27 (4.39)	12.19 (3.79) 12.59 (3.79)		*F* = 6.37, (2,1091), *p* < 0.005	None
SSRS Children Positive Academic Behaviors	Experimental Control	666 826	15,70 (1.18) 15.97 (1.72)	15.90 (1.67) 15.90 (1.67)		*F* = 12.26, (1,1491), *p* < 0.0001		More increases in E boys More decreases in C girls
	Experimental Control	422 574	12.60 (1.60) 12.62 (1.53)	12.76 (1.51) 12.49 (1.59)	12.74 (1.33) 12.72 (1.22)		*F* = 6.84 (2,994), *p* < 0.001	None

When the 1 year follow-up assessments are included in the analysis, reducing the sample size to those who participated in all three assessments, the MANOVA indicates a significant interaction between the three times of measure and the Group [*F* = 17.53 (2,1093), *p* < 0.001] with a mean increase in the Experimental Group from 3.12 (*SD* = 0.37) to 3.30 (*SD* = 0.36) post-test and in the 1 year follow-up to 3.22 (*SD* = 0.37). In the Control Group, the mean of 3.11, *SD* = 0.38 in the pre-test, increased insignificantly in the post-test to mean 3.18, *SD* = 0.37 to return to slightly below the pre-test mean (3.09, *SD* = 0.44) in the 1 year follow-up. There was no significant interaction with sex [*F* = 0.79 (2,1093), NS] or socio-economic level of the school [*F* = 1.87 (1,1093), NS]. Overall, there was a significant interaction between the pre-test and the combined post- test and 1 year follow-up [*F* = 68.69 (1,1093), *p* < 0.0001], with no significant interactions with sex [*F* = 0.46 (1,1093), NS] or with socio-economic level of the school [*F* = 0.12 (1,1093), NS]. Tukey, Scheffe and Bonferroni *post hoc* tests indicated no significant differences between the pre-test, post-test and 1 year follow-up in the Control Group.

#### Social and Academic Skills: The Social Skills Rating System (SSRS)

We conducted exploratory factor analyses in the pre-test responses on data from both the student and teacher versions of the SSRS and did not find the same factors which were identified and used as coding scales in the samples in the United States where the SSRS was initially validated. However, we did identify three factors in the teacher version which each explained over 5% of the variance. The first factor (10.6% of variance), which we labeled ‘Control of Emotions,’ had a very low internal consistency (Cronbach alpha 0.13). The second factor, ‘Positive Academic Behavior’ (7.4% of variance), which included 11 items such as ‘finishes classroom work on time,’ ‘attends to your instructions,’ and ‘ignores peer distractions while doing school work’ had high internal consistency (Cronbach alpha 0.81). The third factor, ‘Initiating Activities’ (5.1% of variance), which included eight items such as ‘Introduces herself or himself to new people without being told,’ also had a high internal consistency (Cronbach alpha 0.88).

In the SSRS Teacher version, there were no significant interactions between Time (pre-test, post-test) and Group (Experimental/Control) in the factors of ‘Control of Emotions’ and ‘Initiating activities.’ There was a significant interaction between Time and Group in ‘Positive Academic Behavior,’ with the Experimental Group increasing from a mean of 12,42 (*SD* = 3.87) to 12.73 (*SD* = 3.97) after participation in the program and the Control Group decreasing in these behaviors (from 12.28, *SD* = 4.31 to *M* = 12.20, *SD* = 4.38) [*F* = 5.35 (1,1475), *p* < 0.02.]. When the 1-year follow-up was included in analyses of the SSRS Teacher version, there was a significant interaction between Time and Group [*F* = 6.37 (2,1091), *p* < 0.005]. There was an overall mean increase in the Experimental Group from pre-test (*M* = 12.45, *SD* = 3.85) to post-test (*M* = 12.66, *SD* = 3.91) followed by a decrease in the 1-year follow-up (*M* = 12.19, *SD* = 3.79); compare to a decrease in the Control Group from pre-test (*M* = 12.37, *SD* = 4.08) to post-test (*M* = 12.27, *SD* = 4.39) and an increase in the 1-year follow-up (*M* = 12.59, *SD* = 3.79).

The same three factors were identified based upon factor analysis in the SSRS Student version. There were no significant interactions between Time (pre-test, post-test) and Group in the factors of ‘Control of Emotions’ [*F* = 0.03 (1,1491), NS] and ‘Initiating’ [*F* = 2.84 (1,1491), NS]. There was a significant interaction between Time and Group in ‘Positive Academic Behavior,’ with the Experimental Group increasing after participation in the program and the Control Group decreasing in these behaviors [*F* = 12.26 (1,1491), *p* < 0.0001] (Experimental Group pre-test mean 15.70, *SD* = 1.18, post-test mean 15.90, *SD* = 1.67; Control Group pre-test mean 15.97, *SD* = 1,72, post-test mean 15.90, *SD* = 1.67). There was no significant interaction with socio-economic level of the school, but there was an interaction between Time, Group and Sex. There were greater mean improvements in boys in the Experimental Group and more decreases in Girls than Boys in the Control Group, but *post hoc* tests did not indicate that the amount of improvements for boys or for girls were significantly different when comparing the Experimental and Control Groups.

When the 1-year follow up of the children’s SSRI was included, there were no significant interactions between Group and Time on ‘Control of Emotions’ [*F* = 0.67 (2,994), NS] and ‘Initiating’ (*F* = 1.59, DF = 2,994, NS). There was a significant interaction between Group and Time on ‘Positive Academic Behavior’ [*F* = 6.84 (2,994), *p* < 0.001] (Experimental Group pre-test mean 12.60, *SD* = 1.6, post-test mean 12.76, *SD* = 1.51, 1-year follow-up mean 12.74, *SD* = 1.33; Control Group pre-test mean 12.62, *SD* = 1.53, post-test mean 12.49, *SD* = 1.59, 1-year follow-up mean 12.72, *SD* = 1.22), with *post hoc* tests indicating a significant increase between the Experimental Group’s pre-test and combined post-test and 1-year follow-up, and a slight non- significant decrease in the combined post-test and 1-year follow-up in the Control Group.

## Discussion

*PSL* was developed in a systematic manner, starting with a needs assessment study and the creation and testing of a pilot program, then testing all new activities for a Beta version and evaluating a revised Beta version. After more revisions and testing of all new and revised activities, the “final” version was developed. This paper reports on the first evaluation of the implementation and effects of *PSL*. The evaluation included detailed assessments of the implementation, qualitative analyses of effects perceived by children, parents and teachers, as well as quantitative research results using a stratified randomized control design.

The first research questions we asked was whether or not PSL could be implemented successfully as planned, and whether children, teachers and parents all enjoyed their participation in the activities. We learned from questionnaires, focus groups and observations in classrooms that the program had high levels of appreciation by everyone involved. The activities could all be implemented as planned by trained teachers, and parents could easily conduct their complementary at-home exercises. We learned, however, that the youngest grade 3 (age 9) children required much more explanations from the teachers, and grade 6 (age 12) children found some of the topics less relevant to their pre-adolescent preoccupations. For this reason, we recommend *PSL* for use in grades 4 and 5 of elementary school, unless more mature content is added to role play activities for grade 6 children, such as dealing with peer pressures to smoke or use of drugs. Currently, in Brazil, the non-profit organization, ASEC-Associação pela Saúde Emocional de Crianças, has modified *PSL* by adding content for adolescents and they are using the modified program with older children in several communities.

We conclude that *PSL* can be conducted as planned, had high levels of participation by children and was well-liked. These positive results may be due to the fact that each activity was carefully tested and refined several times over the course of 5 years. Only activities that could be easily conducted, that children willingly and actively engaged in, and were highly appreciated by children, teachers and parents, were retained in the final iteration of the program.

The main goal of *PSL* is to improve coping skills. Qualitative data indicates that *PSL* was perceived as improving coping skills, based upon reports by children, teachers and parents. We also used several measures of coping in a randomized controlled design, to assess the impact of *PSL.* We included several measures of coping because research suggests that the assessment of coping with children using self-report measures is particularly challenging. Variations in the manner in which questions are asked, and the choice of situations children are asked to tell about, can influence the nature and the quality of the information obtained (Blount et al., 2008). We found that, compared to Control Group children who did not participate in the program, participants in *PSL* had significant increases in the number of coping strategies they said they used in the Kidcope. On the CCQ there were significant increases in the number of coping strategies only when the 1- year follow-up data were included. There were significant increases in the number of things children wrote that they did to improve a situation when they felt sad (Draw and Write), in both the pre-post comparisons and when the 1-year follow-up was included. There also were significant pre-post increases in the number of strategies when asked how they thought people could cope in fictitious situations. These findings indicate that, at least in the short term, *PSL* appears to increase children’s coping abilities, which is the main goal of the program.

There were also significant increases in Emotional Awareness, a key skill for effective coping, and these skills were sustained 1-year later. These significant improvements were present in three of the four hypothetical situations: Victim of Bullying, “Unfair” Parental Restrictions and Witness to Bullying, but not in the situation of the family moving away. This may be due to the fact that the three hypothetical situations where significant effects were observed are fairly common. However, fewer children are likely to have experienced the fourth situation of a family moving away. Therefore, they might not have been able to understand the emotional implications of a move from their personal experiences.

As indicated in both teachers’ observations and children’s responses, there were significant increases in the Experimental group in Positive Academic Behaviors. However, when 1-year follow-up data were included, there was a subsequent decline in both the Experimental and Control Groups to near pre-test levels. The decline the following year occurred when children were in another class with a different teacher who was not familiar with *PSL.* Perhaps, the improvements in Positive Academic Behaviors are specific to contexts where the teacher and the other children in the class have experienced *PSL*, which results in an overall change in relationships and the social environment of the classroom. In a different classroom where not all children experienced *PSL* and the teacher was not knowledgeable about the program content, the classroom environment may no longer have been conducive to sustaining improvements in these behaviors, which were not specifically taught in *PSL*. However, this finding in the quantitative data helps confirm the reports by teachers that their classes were easier to manage, which was seen as a benefit for the teachers of conducting *PSL* in their classes.

The lack of significant differences in the impact of *PSL* on boys and girls is surprising. Originally, the program developers were concerned that boys may be less inclined to talk about feelings and verbalize their concerns, and they were concerned that the comics and exercises may appeal more to boys or girls. It is possible that the repeated testing of the program content on both sexes resulted in developing a final product that is appropriate for both boys and girls. The lack of differences in the program effects for different socio-economic level schools helps validate the usefulness of *PSL* as a universal mental health promotion activity for all children. However, we used general measures of the economic situation in the community. Future research should explore differences between children who are identified as coming from different socio-economic backgrounds to determine if these factors influence the effects of *PSL.*

This study and the systematic iterative development of *PSL* may serve as a model for the development of mental health promotion programs for children. By systematically refining the program content over 5 years and continually evaluating the implementation and effects of the activities, the final version of the program, as this evaluation of the implementation has confirmed, was highly appreciated, had high levels of enthusiastic participation by children, parents and teachers, and had indications of attaining its main goal of improving children’s coping skills. We believe that it is important for all evaluations of program effects to include an evaluation of the program’s implementation to ensure conformity of how the program was delivered with what was expected, and to ensure that levels of participation and appreciation of the program are sufficiently high.

## Conclusion

This study indicates that *Passport: Skills for Life* is a promising mental health promotion program that can increase the abilities of children to cope with difficulties, improve their emotional awareness, and may, at least in the short term, increase positive academic behavior. However, there is a need for more research. Future research should investigate the importance of teacher variables and other potential differences between classrooms and schools using a nested design to assess potential differences in the program impact by classroom and school, which may be associated with variables other than the program implementation. Whenever teachers conduct classroom activities, other factors, such as teacher enthusiasm or skills, may be associated with outcomes. Also, the characteristics of different school environments and populations, other than the general difference in socio-economic level we assessed, may influence the impact of the program on children. Furthermore, although it is costly and difficult to achieve, it is important to confirm if the behavioral changes reported by children and teachers are confirmed by systematic observations of children’s actual behaviors.

Trained teachers can easily conduct this program and *PSL* is well-appreciated by children, teachers and parents. However, it is also important to conduct additional research to assess if its impact is sustained over longer periods of time, as well as studies to ascertain if adaptations need to be made for use in different cultures. Future research could investigate if the program also affects other variables, such as self-esteem and problem behaviors in schools, and if the program may have much longer-term benefits, such as fewer suicidal behaviors later in adolescence. Since *PSL* has the same objectives as the program for first grade children, *Zippy’s Friends*, and *PSL* was conceived as a potential follow-up program for *Zippy’s Friends*, it is important to now determine if using *PSL* with children who have participated in *Zippy’s Friends* produces better or more sustained benefits.

Another important future research goal is to explore what makes this program successful. It would be interesting to know if certain characteristics of the program are keys to its success. One could speculate that the repeated practice of coping skills in games and role pay activities are of crucial importance, or that teachers motivation levels play an important role. These and other hypotheses could be verified, for example, by assessing variants of the program, or comparing results with teachers who have different levels of enthusiasm.

*PSL* can be successful implemented as planned and is highly appreciated by children, teachers and parents. We would expect that this is due to the 5 year process of perfecting the program with continued evaluation activities. Our evaluation of the effects indicates that, compared to the control group, participants use more helpful coping strategies, which was the main goal of *PSL.* Participants also had increased emotional awareness, and in the year of the program, they had more positive academic behaviors. We conclude that this is a promising mental health promotion program. However, additional evaluation studies should be undertaken, particularly of its effects with children who previously had the program for younger children, *Zippy’s Friends.* One could speculate that a combination of both programs, which focus on improving coping skills, should result in more sustained use of helpful coping strategies. Also, longer term longitudinal research should be undertaken to determine if benefits from the program are sustained over longer periods of time.

## Data Availability Statement

The raw data supporting the conclusions of this article will be made available by the authors, without undue reservation.

## Ethics Statement

The studies involving human participants were reviewed and approved by Comité institutionnel d’éthique de la recherche avec les êtres humains of the Université du Québec à Montréal and the Université de Montréal. Written informed consent to participate in this study was provided by the participants’ legal guardian/next of kin.

## Author Contributions

All authors listed have made a substantial, direct and intellectual contribution to the work, and approved it for publication.

## Conflict of Interest

The authors declare that the research was conducted in the absence of any commercial or financial relationships that could be construed as a potential conflict of interest.

## Footnotes

1 www.passportskillsforlife.ca
